# Association between stress hyperglycemia ratio and acute kidney injury development in patients with sepsis: a retrospective study

**DOI:** 10.3389/fendo.2025.1542591

**Published:** 2025-04-15

**Authors:** Yipeng Fang, Aizhen Dou, Ying Zhang, Hui Xie, Yunfei Zhang, Yan Cui, Keliang Xie

**Affiliations:** ^1^ Department of Critical Care Medicine, Tianjin Medical University General Hospital, Tianjin, China; ^2^ Firth Clinical College, Xinxiang Medical University, Xinxiang, Henan, China; ^3^ Tianjin Hospital, Tianjin, China; ^4^ Department of Pathogen Biology, School of Basic Medical Sciences, Tianjin Medical University, Tianjin, China; ^5^ Department of Anesthesiology, Tianjin Institute of Anesthesiology, Tianjin Medical University General Hospital, Tianjin, China

**Keywords:** sepsis, sepsis-associated acute kidney injury, stress hyperglycemia, stress hyperglycemia ratio, biomarker

## Abstract

**Background:**

Stress hyperglycemia ratio (SHR), which adjusts blood glucose levels using glycated hemoglobin to eliminate the influence of chronic hyperglycemia, has been demonstrated to have superior predictive value than absolute hyperglycemia. However, its predictive value for sepsis-associated acute kidney injury (SA-AKI) remains unclear. This study aimed to investigate the relationship between the SHR and the risk of developing SA-AKI.

**Methods:**

Data were extracted from the Medical Information Mart for Intensive Care IV (MIMIC-IV) database. Restricted cubic splines (RCS) were employed to depict the relationship between SHR and the likelihood of SA-AKI, determining an optimal cut-off value. Based on this threshold, patients were categorized into two groups. Logistic regression was utilized to evaluate SHR’s predictive value for SA-AKI, with adjustments for confounding variables. Propensity score matching (PSM) was applied to balance baseline characteristics. Subgroup and sensitivity analyses were conducted.

**Results:**

A total of 2,249 patients were included. The RCS curve indicated a non-linear positive association between SHR and the likelihood of SA-AKI (*P* for non-linearity < 0.001), with an optimal cut-off at 1.55. Accordingly, patients were divided into SHR ≤ 1.55 and SHR > 1.55 subgroups, comprising 1,131 and 1,118 individuals, respectively. A higher incidence of SA-AKI was observed in the SHR > 1.55 group (38.64% vs. 27.23%, *P* < 0.001). This association persisted after baseline adjustment through PSM. Logistic regression analysis confirmed that SHR > 1.55 was linked to increased odds of SA-AKI in both unadjusted (OR: 1.68, *P* < 0.001) and adjusted models (OR: 1.73, *P* < 0.001), with SHR ≤ 1.55 serving as the reference. In subgroup analysis, all subgroups consistently demonstrated a significant association between SHR > 1.55 and elevated odds of SA-AKI (all OR > 1). Sensitivity analysis validated that SHR > 1.55 remained significantly correlated with SA-AKI occurrence in the survival subgroup (OR: 1.46, *P* < 0.001) and the non-CKD subgroup (OR: 1.69, *P* < 0.001).

**Conclusion:**

The findings indicate a non-linear positive relationship between SHR and the likelihood of SA-AKI in patients with sepsis, suggesting that SHR could be a potential predictor for SA-AKI.

## 1 Background

Sepsis is a complex condition triggered by infection, leading to a cascade of pathological, physiological, and molecular changes in the body (1). It remains a leading cause of mortality in intensive care units (ICUs) worldwide (2), often associated with the onset of multi-organ failure. When acute kidney injury (AKI) develops in the context of sepsis, it is referred to as sepsis-associated acute kidney injury (SA-AKI) (3, 4). Evidence indicates that the clinical outcomes for SA-AKI are poorer compared to sepsis accompanied by other complications (3, 5), underscoring the potential benefits of early SA-AKI detection to improve the prognosis of patients with sepsis.

Stress hyperglycemia frequently occurs in acute illnesses, characterized by a transient rise in blood glucose levels due to inflammatory and neuroendocrine disturbances during the illness (6). However, assessing stress hyperglycemia based solely on blood glucose levels may fail to account for the effects of chronic hyperglycemia. To address this limitation, glycated hemoglobin (HbA1c) has been suggested as a baseline indicator of blood glucose, with the stress hyperglycemia ratio (SHR) proposed as a more precise biomarker for evaluating acute hyperglycemia rather than absolute blood glucose elevation (7). SHR was initially reported by Roberts GW in 2015 and is calculated by dividing the absolute blood glucose level by the estimated chronic glucose level derived from HbA1c conversion (7). HbA1c reflects average glucose levels over the previous three months and remains relatively unaffected by the acute onset of illness (8–10). Previous research has predominantly explored the association between SHR and adverse outcomes in cardiovascular events, such as acute coronary syndrome and severe myocardial infarction (11, 12).

During sepsis, the body undergoes a state of stress characterized by activation of the sympathetic nervous system and elevated corticosteroid levels, resulting in increased glucose metabolism and dysregulation, which in turn contribute to higher mortality rates (13–15). Current research examining the link between stress hyperglycemia and sepsis is still limited. In 2020, Fabbri et al. conducted an observational study, finding that SHR ≥ 1.14 was strongly associated with all-cause mortality (OR = 5.25, 95% CI 3.62–7.63) among patients with diabetes and sepsis (16). Another study demonstrated a U-shaped relationship between SHR and both 28-day all-cause mortality and in-hospital mortality in patients with sepsis, regardless of type 2 diabetes status (17). Evidence suggests that hyperglycemia at admission may impair renal function by intensifying oxidative stress, inflammation, endothelial dysfunction, hypovolemia, and dehydration, all of which reduce renal perfusion (6). Research by Yu Shan et al. showed a significant correlation between SHR and increased incidence of contrast-induced AKI (CI-AKI) in patients undergoing coronary angiography (CAG) or percutaneous coronary intervention (PCI) (18). However, no studies to date have investigated the potential association between SHR and AKI occurrence specifically in patients with sepsis.

This study hypothesized that higher SHR is associated with increased likelihood of developing SA-AKI, indicating its potential value for early identification of SA-AKI. The aim was to explore the relationship between SHR and the likelihood of AKI in patients with sepsis, with the goal of informing early detection and prevention strategies, ultimately leading to improving patient outcomes.

## 2 Study design and methods

### 2.1 Data source

This single-center retrospective study leveraged data from the Medical Information Mart for Intensive Care (MIMIC-IV version 2.2) database, a comprehensive, publicly accessible dataset containing over 70,000 de-identified ICU records from 50,000 patients admitted to Beth Israel Deaconess Medical Center (BIDMC) between 2008 and 2019 (19). Access to the database was granted following completion of training and examination through the National Institutes of Health (NIH) online course (certificate number 43025968). The MIMIC-IV database has ethical approval from the Massachusetts Institute of Technology (MIT) Ethics Committee, and the requirement for informed consent was waived due to the retrospective nature of the study and the use of anonymized data.

### 2.2 Study population

The study population consisted of patients admitted to the ICU from 2008 to 2019, with the following exclusion criteria applied: repeat admissions, patients under 18 years of age, absence of sepsis diagnosis, AKI preceding the onset of sepsis, incomplete SHR data (missing blood glucose or HbA1c), and ICU stays shorter than 48 hours. Sepsis was diagnosed according to the Sepsis 3.0 criteria (20), while sepsis-associated acute kidney injury (SA-AKI) was defined based on the 28th Acute Disease Quality Initiative workgroup’s consensus, as AKI meeting the KDIGO diagnostic criteria following sepsis onset (21). In this study, only the creatinine-based KDIGO criteria were utilized, with AKI defined as either a 50% increase in serum creatinine from baseline within seven days of sepsis diagnosis or an increase in serum creatinine by more than 26.5 μmol/L within 48 hours (22).

### 2.3 Exposure and outcomes

The primary exposure variable was the SHR, a composite index derived from blood glucose and HbA1c, calculated using the following formula: SHR = blood glucose (mg/dL)/(28.7 × HbA1c (%) − 46.7) (7). The highest recorded blood glucose and HbA1c values during the ICU stay were used for SHR calculation in patients with sepsis.

The primary outcome was the incidence of new-onset AKI within the first 48 hours following sepsis diagnosis. Secondary outcomes included hospital mortality, ICU mortality, 28-day mortality, 90-day mortality, as well as length of hospital stay (hospital-LOS) and ICU stay (ICU-LOS).

### 2.4 Data extraction and definitions

In this study, baseline information included demographic data, comorbidities, laboratory parameters, mean blood pressure (MBP), interventions, and disease severity scores. Demographic details covered age, sex, race, and body weight. Comorbidities were identified using International Classification of Diseases (ICD) codes and the Charlson Comorbidity Index, including conditions such as coronary heart disease, heart failure, hypertension, diabetes mellitus, anemia, chronic pulmonary disease, chronic kidney disease, liver disease, and malignant cancer. Laboratory data collected from ICU admission until sepsis diagnosis included the maximum or minimum values of white blood cell count, hemoglobin, mean corpuscular volume (MCV), red cell distribution width (RDW), platelets, sodium, potassium, total calcium, anion gap (AG), alanine aminotransferase (ALT), aspartate aminotransferase (AST), alkaline phosphatase (ALP), bilirubin, creatinine, blood urea nitrogen (BUN), international normalized ratio (INR), prothrombin time (PT), activated partial thromboplastin time (APTT), lactate, and SHR. Data on interventions received from ICU admission to sepsis diagnosis, such as mechanical ventilation, vasoactive drugs, and diuretic use, were also collected. Vital signs within the same timeframe were recorded.

### 2.5 Data cleaning

Patients missing either blood glucose or HbA1c data required for SHR calculation were excluded from the final analysis. To preserve data integrity in AKI diagnosis, all creatinine values were maintained in their original form without any imputation. Outliers for continuous variables with normal distribution were defined as values more than three standard deviations (SD) from the mean. For non-normally distributed continuous variables, outliers were defined as values beyond three times the interquartile range (IQR) above the third quartile (Q3) or below the first quartile (Q1) and were treated as missing data. Missing values were imputed using the mean or median, depending on data distribution, if the percentage of missing data was below 10%. For 10-20% missing data, random forest interpolation was applied. Variables with over 20% missing values were excluded from the analysis.

### 2.6 Statistical analysis

Continuous variables following a normal distribution were presented as mean ± SD and analyzed using the unpaired Student’s t-test. Those with a non-normal distribution were reported as median and IQR and assessed with the Mann-Whitney U test. Categorical variables were expressed as counts and percentages, with analyses performed using the Chi-square test.

Restricted cubic spline (RCS) regression was employed to visualize the potential non-linear relationship between SHR and the odds of developing SA-AKI. An optimal cut-off value of 1.55 was identified, prompting the categorization of patients into SHR ≤ 1.55 and SHR > 1.55 groups. Logistic regression analysis, both univariate and multivariate, was used to investigate the association between SHR and SA-AKI odds. Adjusted models were constructed to minimize the influence of confounders, incorporating demographic characteristics and comorbidities. Multicollinearity was assessed using the variance inflation factor (VIF), with variables showing VIF > 10 converted to binary variables based on median or mean values to address collinearity concerns. Adjusted models were also applied in the RCS analysis. Propensity score matching (PSM) was conducted to balance baseline characteristics, employing a 1:1 nearest neighbor matching without replacement and a caliper width of 0.02. Subgroup analyses were performed based on age, sex, race, weight, diabetes status, SOFA score, and diuretic use to explore potential interactions and assess result robustness. Forest plots were generated to illustrate subgroup analysis findings. Additionally, sensitivity analysis was performed by excluding patients who died or had a prior history of CKD to further validate the stability of the results.

Data analyses were conducted using Stata (version 15.0 S.E.) and R (version 4.3.2), with statistical significance set at a two-sided *P*-value of ≤ 0.05.

## 3 Results

### 3.1 Baseline information and clinical endpoints

**Figure 1:**
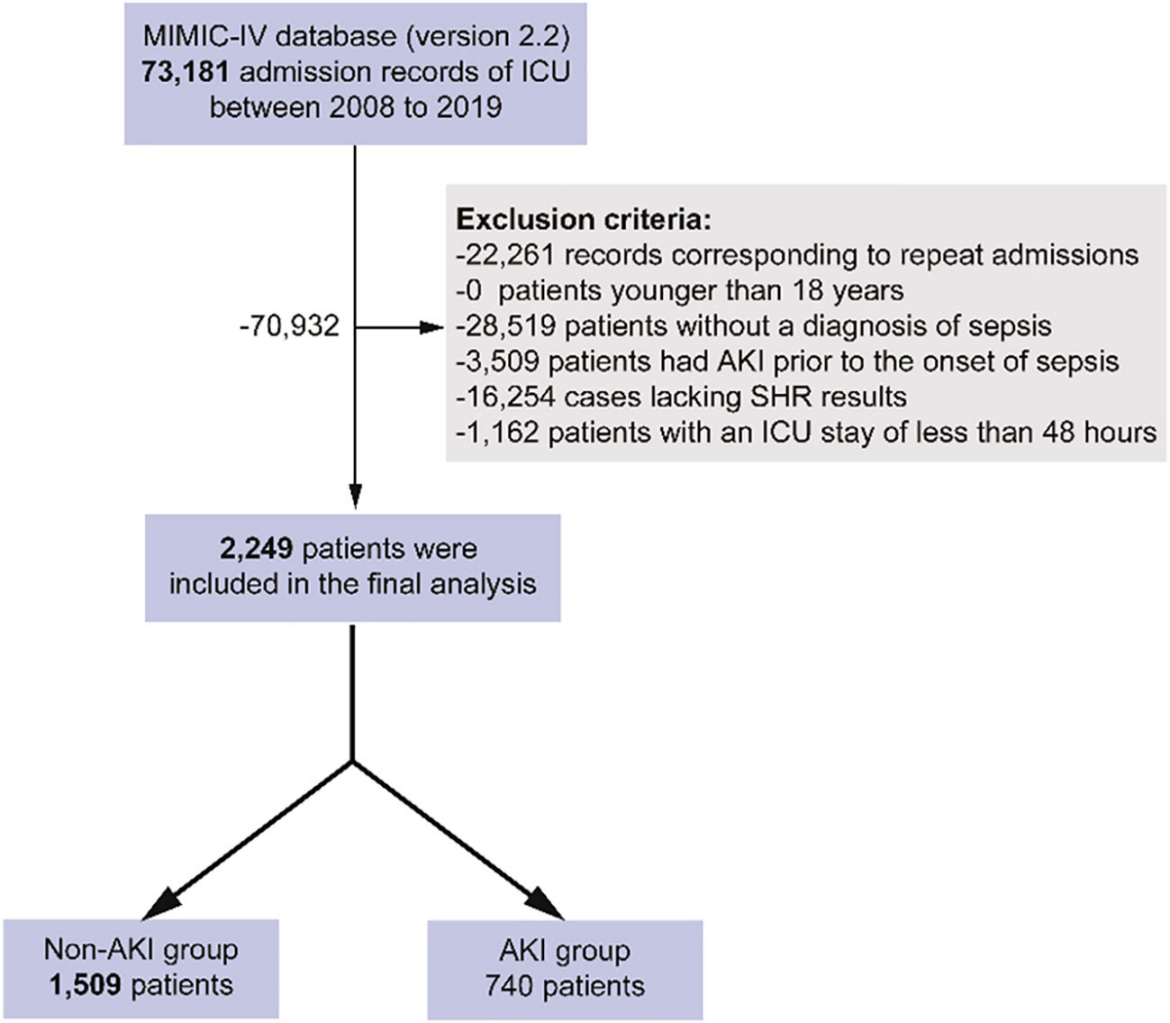
Flowchart of patient selection.

**Figure 1** illustrates the flowchart for patient selection, showing that 2,249 patients were included in the study, with 740 patients diagnosed with SA-AKI and 1,509 without. **Table 1** compares baseline characteristics between the SA-AKI and non-SA-AKI groups. Patients in the SA-AKI group were older (70.14 ± 13.09 vs. 66.71 ± 14.60, *P* < 0.001) and had a higher prevalence of coronary artery disease, heart failure, diabetes, anemia, and chronic kidney disease, while hypertension was less common (all *P* < 0.01). Additionally, patients with SA-AKI were more likely to require mechanical ventilation, vasoactive drugs, and diuretics and had elevated SOFA and SAPSII scores (all *P* < 0.01). Laboratory comparisons revealed that the SA-AKI group had significantly higher levels of white blood cells, RDW, AG, creatinine, BUN, INR, PT, APTT, lactate, and SHR, while hemoglobin, platelet count, and sodium levels were lower (all *P* < 0.001). The SA-AKI group also exhibited higher mortality rates and longer LOS compared to the non-SA-AKI group (all *P* < 0.01).

**Table 1:** Baseline information and clinical endpoints in patients with and without SA-AKI.

Variable	Overall (N = 2249)	Non-SAAKI (N = 1509)	SA-AKI (N = 740)	*P*-value
Age (years)	67.84 ± 14.21	66.71 ± 14.60	70.14 ± 13.09	< 0.001
Male (%)	1367 (60.78)	896 (59.38)	471 (63.65)	0.051
Ethnicity, white (%)	1300 (57.80)	855 (56.66)	445 (60.14)	0.117
Weight (kg)	84.86 ± 23.38	84.60 ± 23.26	85.40 ± 23.64	0.443
Comorbidity				
Coronary heart disease (%)	1025 (45.58)	597 (39.56)	428 (57.84)	< 0.001
Heart failure (%)	787 (34.99)	455 (30.15)	332 (44.86)	< 0.001
Hypertension (%)	1108 (49.27)	802 (53.15)	306 (41.35)	< 0.001
Diabetes mellitus (%)	882 (39.22)	556 (36.85)	326 (44.05)	0.001
Anemia (%)	1066 (47.40)	629 (41.68)	437 (59.05)	< 0.001
Chronic pulmonary disease (%)	596 (26.50)	386 (25.58)	210 (28.38)	0.158
Chronic kidney disease (%)	413 (18.36)	173 (11.46)	240 (32.43)	< 0.001
Liver disease (%)	244 (10.85)	155 (10.27)	89 (12.03)	0.209
Malignant cancer (%)	142 (6.31)	104 (6.89)	38 (5.14)	0.107
Laboratory parameter				
White blood cell (k/uL)	12.0 (9.1,16.1)	11.7 (8.9,15.5)	12.6 (9.3,17.6)	< 0.001
Hemoglobin (g/dL)	10.57 ± 1.97	10.71 ± 1.91	10.28 ± 2.04	< 0.001
MCV (fl)	90.86 ± 6.50	90.87 ± 6.58	90.86 ± 6.36	0.987
RDW (%)	14.2 (13.4,15.4)	14.1 (13.3,15.2)	14.5 (13.6,15.7)	< 0.001
Platelets (k/uL)	170 (127,228)	174 (134,233)	159 (118,218)	< 0.001
Sodium (mmol/L)	138.97 ± 4.35	139.22 ± 4.44	138.46 ± 4.12	< 0.001
Potassium (mmol/L)	4.17 ± 0.60	4.09 ± 0.57	4.34 ± 0.62	< 0.001
Total calcium (mmol/L)	8.35 ± 0.68	8.35 ± 0.66	8.35 ± 0.72	0.980
AG (mmol/L)	13 (11,16)	13 (11,15)	14 (11,17)	< 0.001
ALT (IU/L)	28 (17,50)	29 (18,49)	27 (17,53)	0.314
AST (IU/L)	37 (23,70)	37 (23,67)	36 (21,80)	0.873
ALP (IU/L)	75 (59,96)	75 (60,94)	76 (57,99)	0.786
Bilirubin (mg/dL)	0.6 (0.4,0.9)	0.6 (0.4,0.9)	0.6 (0.4,1.0)	0.203
Creatinine (mg/dl)	0.9 (0.7,1.3)	0.9 (0.7,1.1)	1.2 (0.9,1.7)	< 0.001
BUN (mg/dL)	18 (13,27)	16 (12,24)	22 (16,34)	< 0.001
INR	1.35 ± 0.40	1.32 ± 0.37	1.41 ± 0.45	< 0.001
PT (s)	13.8 (12.6,15.5)	13.7 (12.4,15.2)	14.3 (13.1,16.3)	< 0.001
APTT (s)	30.4 (27.2,36.3)	30.1 (26.7,34.9)	31.7 (28.4,39.9)	< 0.001
Lactate (mmol/L)	1.8 (1.3,2.4)	1.7 (1.3,2.2)	2.0 (1.4,2.9)	< 0.001
SHR	1.62 ± 0.43	1.59 ± 0.41	1.69 ± 0.46	< 0.001
Mean blood pressure (mmHg)	78.17 ± 10.33	79.65 ± 10.62	75.15 ± 8.99	< 0.001
Intervention				
Mechanical ventilation (%)	1472 (65.45)	929 (61.56)	543 (73.38)	< 0.001
Vasoactive drug (%)	819 (36.42)	466 (30.88)	353 (47.70)	< 0.001
Diuretic exposure (%)	1225 (54.47)	788 (52.22)	437 (59.05)	0.002
Disease severity score				
SOFA score	6 (4,9)	5 (4,8)	7 (5,10)	< 0.001
SAPSII score	37 (31,45)	36 (29,42)	41 (34,51)	< 0.001
Outcomes				
Hospital mortality (%)	220 (9.78)	122 (8.08)	98 (13.24)	< 0.001
ICU mortality (%)	174 (7.74)	89 (5.90)	85 (11.49)	< 0.001
28-day mortality (%)	289 (12.85)	174 (11.53)	115 (15.54)	0.008
90-day mortality (%)	398 (17.65)	238 (15.77)	159 (21.49)	0.001
Hospital LOS (days)	11.5 (7.5,17.8)	11.1 (7.3,17.1)	12.0 (7.9,19.2)	0.009
ICU LOS (days)	4.8 (3.0,8.6)	4.5 (2.9,8.3)	5.1 (3.2,8.9)	0.002

Tip: Continuous variables are presented as mean (standard deviation) or median (interquartile range), and categorical variables are expressed as count (percentage). MCV, erythrocyte mean corpuscular volume; RDW, red blood cell distribution width; AG, anion gap; ALT, glutamic-pyruvic transaminase; AST, glutamic oxaloacetic transaminase; ALP, alkaline phosphatase; BUN, blood urea nitrogen; INR, international normalized ratio; PT, prothrombin time; APTT, activated partial thromboplastin time; SHR, Stress hyperglycemia ratio; SOFA, Sequential Organ Failure Assessment; SAPS, Simplified Acute Physiology Score; ICU, intensive care unit; LOS, length of stay.

### 3.2 Non-linear relationship between SHR and the odds of SA-AKI

**Figure 2:**
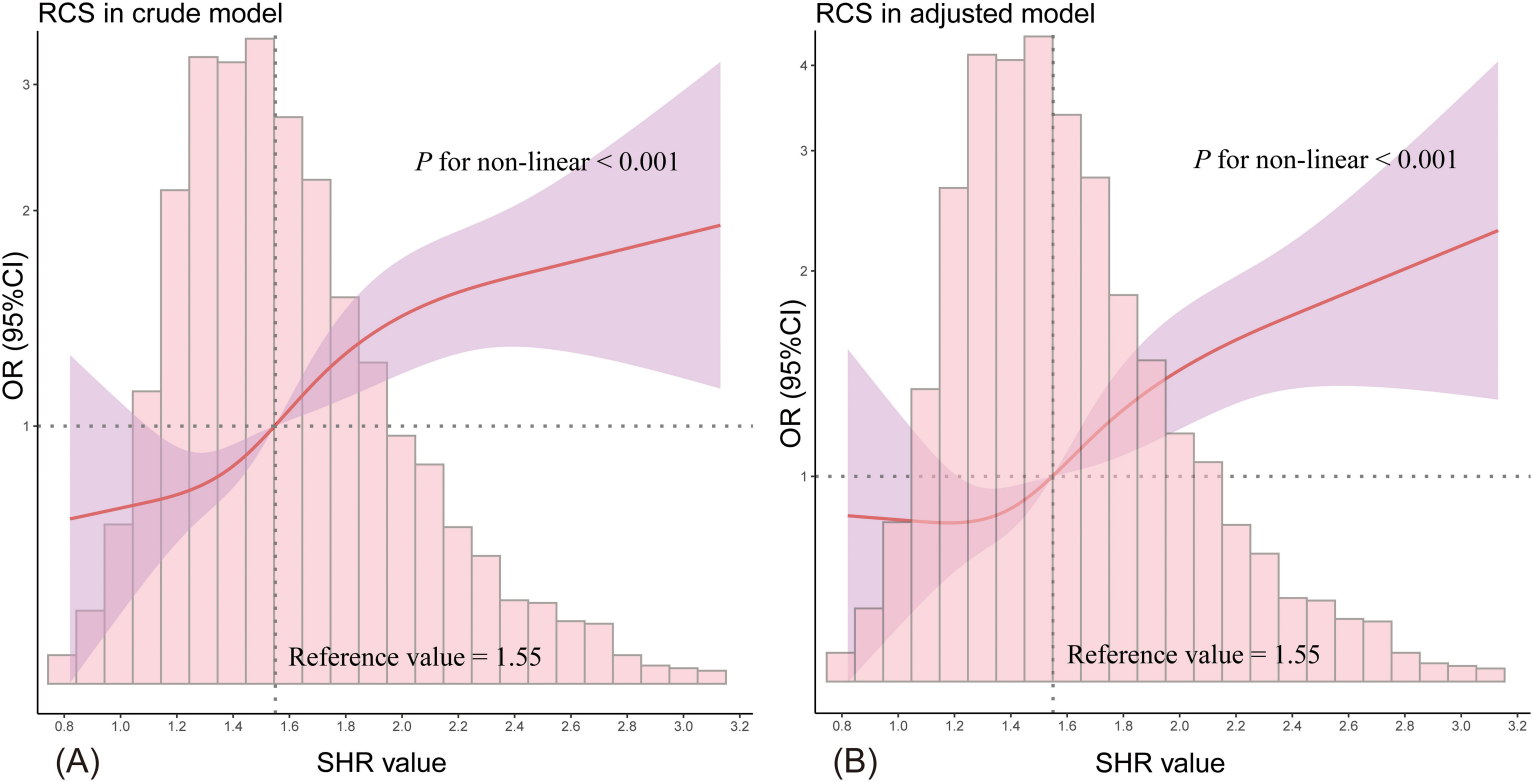
The RCS curve illustrates the non-linear relationship between SHR and the likelihood of SA-AKI. **(A)** shows the results from the unadjusted model, while **(B)** presents the adjusted model outcomes. The red solid line represents the odds ratio (OR), the purple shaded area indicates the 95% confidence intervals (95% CI), and the horizontal dashed line marks the null line (OR = 1). The accompanying bar chart displays the distribution of patient proportions.

**Figure 2** displays the RCS curve analysis, revealing a non-linear positive relationship between SHR and the likelihood of developing SA-AKI (*P* for non-linearity < 0.001), with the odds increasing as SHR rises. An SHR value above 1.55 emerged as an independent risk factor for SA-AKI (**Figure 2A**), a pattern that persisted in the adjusted model (**Figure 2B**).

### 3.3 Clinical outcomes across various SHR categories

Patients with SHR > 1.55 had a significantly higher incidence of SA-AKI (38.64% vs. 27.23%, *P* < 0.001, **Table 2**) compared to those with SHR ≤ 1.55. However, no significant difference was found in the stage of SA-AKI between the groups (*P* = 0.214). More patients with SA-AKI in the SHR > 1.55 group required CRRT treatment than those in the SHR ≤ 1.55 group (*P* = 0.006). Additionally, the SHR > 1.55 group showed significantly higher 28-day, 90-day, hospital, and ICU mortality rates, as well as longer hospital and ICU LOS (all *P* < 0.001). Patients with SHR > 1.55 also had higher SOFA scores and were more likely to need mechanical ventilation and vasopressor support (all *P* < 0.001). After adjusting for baseline characteristics using PSM, SHR > 1.55 remained significantly associated with a higher incidence of SA-AKI (37.83% vs. 28.01%, *P* < 0.001), as detailed in **Table 3**.

**Table 2:** Clinical outcomes across various SHR categories.

	Overall (N = 2249)	SHR ≤ 1.55 (N = 1131)	SHR > 1.55 (N = 1118)	*P*-value
SA-AKI (%)	740 (32.90)	308 (27.23)	432 (38.64)	< 0.001
SA-AKI stage				0.214
Stage 1 (%)	599 (80.95)	255 (82.79)	344 (79.63)	
Stage 2 (%)	88 (11.89)	37 (12.01)	51 (11.81)	
Stage 3 (%)	53 (7.16)	16 (5.19)	37 (8.56)	
CRRT for SA-AKI (%)	60 (8.81)	15 (4.87)	45 (10.42)	0.006
28-day mortality (%)	289 (12.85)	113 (9.99)	176 (15.74)	< 0.001
90-day mortality (%)	397 (17.65)	143 (12.64)	254 (22.72)	< 0.001
hospital mortality (%)	220 (9.78)	77 (6.81)	143 (12.79)	< 0.001
ICU mortality (%)	174 (7.74)	51 (4.51)	123 (11.00)	< 0.001
Hospital LOS (days)	11.5 (7.5,17.8)	9.7 (6.7,14.9)	13.7 (8.8,21.5)	< 0.001
ICU LOS (days)	4.8 (3.0,8.6)	4.0 (2.7,6.3)	6.0 (3.4,11.3)	< 0.001
SOFA score	6 (4,9)	5 (4,8)	7 (5,10)	< 0.001
Mechanical ventilation (%)	1472 (65.45)	696 (61.54)	776 (69.41)	< 0.001
Vasoactive drug (%)	819 (36.42)	318 (28.12)	501 (44.81)	< 0.001

Tip: Continuous variables are presented as mean (standard deviation) or median (interquartile range), and categorical variables are expressed as count (percentage); SHR, Stress hyperglycemia ratio; SA-AKI, Sepsis-associated acute kidney injury; CRRT, continuous renal replacement therapy; ICU, intensive care unit; LOS, length of stay; SOFA, Sequential Organ Failure Assessment.

**Table 3:** Baseline information and clinical endpoints in PSM cohorts.

Variable	SHR ≤ 1.55 (N = 896)	SHR > 1.55 (N = 896)	*P*-value
Age (years)	68.07 ± 14.82	68.23 ± 13.77	0.809
Male (%)	537 (59.93)	544 (60.71)	0.735
Ethnicity, white (%)	514 (57.37)	519 (57.92)	0.811
Weight (kg)	84.49 ± 23.49	84.87 ± 22.95	0.728
Comorbidity			
Coronary heart disease (%)	396 (44.20)	413 (46.09)	0.420
Heart failure (%)	311 (34.71)	305 (34.04)	0.765
Hypertension (%)	434 (48.44)	448 (50.00)	0.508
Diabetes mellitus (%)	351 (39.17)	336 (37.50)	0.466
Anemia (%)	420 (46.88)	416 (46.43)	0.850
Chronic pulmonary disease (%)	240 (26.79)	236 (26.34)	0.831
Chronic kidney disease (%)	165 (18.42)	159 (17.75)	0.713
Liver disease (%)	76 (8.48)	67 (7.48)	0.433
Malignant cancer (%)	60 (6.70)	52 (5.80)	0.435
Outcome indicators			
SA-AKI (%)	251 (28.01)	339 (37.83)	< 0.001
SA-AKI stage			0.828
Stage 1 (%)	205 (81.67)	274 (80.83)	
Stage 2 (%)	30 (11.95)	39 (11.50)	
Stage 3 (%)	16 (6.37)	26 (7.67)	
CRRT for SA-AKI (%)	15 (5.98)	28 (8.26)	0.291
28-day mortality (%)	101 (11.27)	127 (14.17)	0.065
90-day mortality (%)	129 (14.40)	184 (20.54)	0.001
hospital mortality (%)	68 (7.59)	100 (11.16)	0.010
ICU mortality (%)	45 (5.02)	87 (9.71)	< 0.001
Hospital LOS (days)	10.0 (6.9,15.2)	13.1 (8.6,20.9)	< 0.001
ICU LOS (days)	4.1 (2.8,6.7)	5.8 (3.3,11.3)	< 0.001
SOFA score	5 (4,8)	7 (4,9)	< 0.001
Mechanical ventilation (%)	543 (60.60)	630 (70.31)	< 0.001
Vasoactive drug (%)	263 (29.35)	383 (42.75)	< 0.001

Tip: Continuous variables are presented as mean (standard deviation) or median (interquartile range), and categorical variables are expressed as count (percentage); SA-AKI, sepsis-associated acute kidney injury; CRRT, continuous renal replacement therapy; ICU, intensive care unit; LOS, length of stay; SOFA, Sequential Organ Failure Assessment; SHR, Stress hyperglycemia ratio; PSM, Propensity scores for patients were calculated using a multivariate logistic regression model. To equalize baseline information, a 1:1 nearest neighbor matching without replacement was utilized, employing a caliper setting of 0.02.

### 3.4 Logistic regression analysis

**Table 4** presents the logistic regression analysis results, indicating that when SHR was analyzed as a continuous variable, it was significantly associated with a higher likelihood of developing SA-AKI (OR 1.76, 95% CI 1.44–2.15, *P* < 0.001). This association remained robust after adjusting for potential confounders (OR 1.94, 95% CI 1.56–2.40, *P* < 0.001). Using SHR ≤ 1.55 as a reference, SHR > 1.55 was significantly linked to the incidence of SA-AKI (OR 1.68, 95% CI 1.41–2.01, *P* < 0.001), indicating a 73% increased probability of SA-AKI (OR 1.73, 95% CI 1.44–2.09, *P* < 0.001). Additionally, SHR was categorized into quartiles: Q1 (SHR ≤ 1.31), Q2 (1.31 < SHR ≤ 1.55), Q3 (1.55 < SHR ≤ 1.85), and Q4 (SHR > 1.85). Compared to Q1, the Q4 category showed an 82% higher likelihood of SA-AKI (OR 1.82, 95% CI 1.42–2.35, *P* < 0.001), followed by Q3 (OR 1.58, 95% CI 1.22–2.03, *P* < 0.001). No significant difference was observed between Q1 and Q2 (OR 1.02, 95% CI 0.78–1.32, *P* = 0.908).

**Table 4:** Logistic regression analysis evaluating the impact of SHR on the development of SA-AKI.

	Unadjusted Model		Adjusted Model	
	Crude OR (95% CI)	*P* value	Adjusted OR (95% CI)	*P* value
Continuous				
SHR increases by one unit	1.76 (1.44-2.15)	< 0.001	1.94 (1.56-2.40)	< 0.001
Category				
SHR ≤ 1.55	**Reference**			
SHR > 1.55	1.68 (1.41-2.01)	< 0.001	1.73 (1.44-2.09)	< 0.001
Quartering				
Q1 (SHR ≤ 1.31)	**Reference**			
Q2 (1.31 < SHR ≤ 1.55)	1.02 (0.78-1.32)	0.908	0.98 (0.75-1.29)	0.887
Q3 (1.55 < SHR ≤ 1.85)	1.58 (1.22-2.03)	< 0.001	1.50 (1.15-1.96)	0.003
Q4 (SHR > 1.85)	1.82 (1.42-2.35)	< 0.001	1.96 (1.51-2.56)	< 0.001

Adjusted Model: Adjusted for sex, age, body weight, ethnicity, and comorbidities (hypertension, coronary heart disease, heart failure, diabetes, chronic kidney disease, chronic pulmonary disease, liver disease, anemia, malignant cancer). SHR, Stress hyperglycemia ratio; SA-AKI, Sepsis-associated acute kidney injury; CI confidence interval; OR: odds ratio.

### 3.5 Subgroup analysis

**Figure 3:**
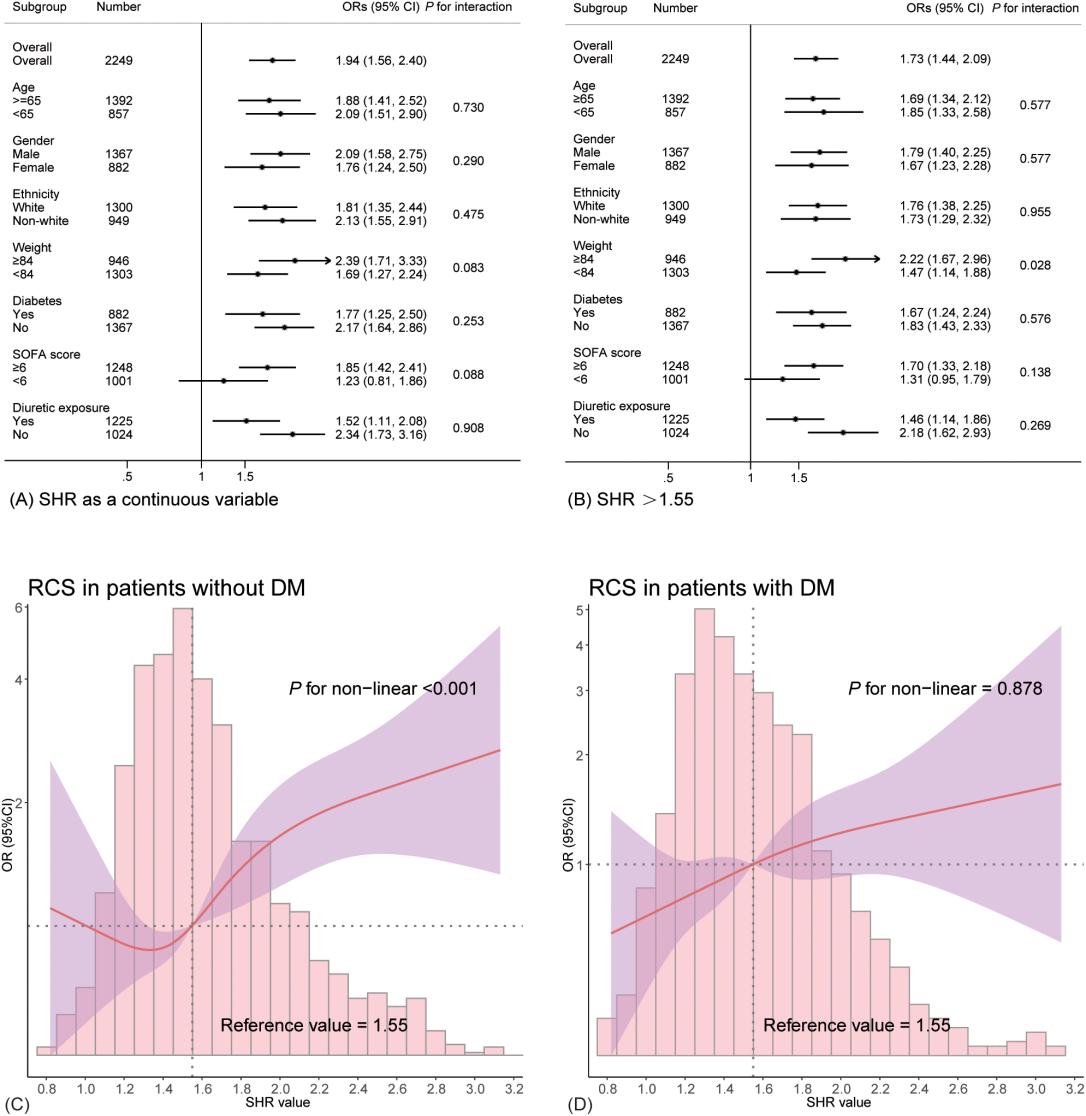
Subgroup analysis depicted in forest plots and RCS curves. **(A)** presents the results with SHR as a continuous variable, while **(B)** shows the findings for SHR >1.55. **(C, D)** illustrates the relationship between SHR and the likelihood of SA-AKI in non-diabetic and diabetic patients.

**Figures 3A, B** depicts the forest plot from the subgroup analysis. Apart from a significant interaction between body weight and SHR (*P* for interaction = 0.028), no other significant interactive effects were found, including diabetes (all *P* > 0.05). Obese patients (body weight ≥ 84 kg) exhibited a more pronounced association with SA-AKI (OR 2.22, 95% CI 1.67–2.96). The analysis consistently demonstrated a significant relationship between elevated SHR and increased odds of SA-AKI (all OR > 1). To further explore how diabetes affects the relationship between SHR and developing SA-AKI, RCS curves were constructed in both diabetic and non-diabetic populations separately. While 1.55 remained a valid cutoff, and the non-diabetic subgroup showed a nonlinear relationship consistent with the overall cohort (*P* for non-linearity < 0.001, see in **Figure 3C**). However, the diabetic subgroup exhibited a linear relationship with a weaker association between SHR and SA-AKI risk (*P* for non-linearity = 0.878, shown in **Figure 3D**).

### 3.6 Sensitivity analysis

Further logistic regression was performed after excluding non-survivors and patients with a history of chronic kidney disease (CKD) to strengthen the robustness of the findings (**Table 5**). The results showed that, when treated as a continuous variable, SHR remained significantly associated with higher odds of developing SA-AKI in both the survival subgroup (OR 1.60, 95% CI 1.25–2.04, *P* < 0.001) and the non-CKD subgroup (OR 2.02, 95% CI 1.59–2.57, *P* < 0.001). When SHR was treated as a categorical variable, the significant association persisted (all *P* < 0.001). Using Q1 as the reference, Q4 continued to show a significant association with increased SA-AKI odds (*P* < 0.001), while no significant differences were observed in Q3 and Q2 (all *P* > 0.05).

**Table 5:** Sensitivity analysis in survival and non-CKD subgroups.

	Survival subgroup (N = 1852)		Non-CKD subgroup (N = 1836)	
	Adjusted OR (95% CI)	*P*-value	Adjusted OR (95% CI)	*P*-value
Continuous				
SHR increases by one unit	1.60 (1.25-2.04)	< 0.001	2.02 (1.59-2.57)	< 0.001
Category				
SHR ≤ 1.55	Reference			Reference
SHR > 1.55	1.46 (1.19-1.80)	< 0.001	1.69 (1.37-2.09)	< 0.001
Quarter				
Q1 (SHR ≤ 1.31)	Reference			Reference
Q2 (1.31 < SHR ≤ 1.55)	0.89 (0.67-1.19)	0.446	0.90 (0.66-1.22)	0.488
Q3 (1.55 < SHR ≤ 1.85)	1.29 (0.97-1.72)	0.086	1.30 (0.96-1.77)	0.091
Q4 (SHR > 1.85)	1.49 (1.11-2.00)	< 0.001	1.93 (1.43-2.60)	< 0.001

Adjusted Model: Adjusted for sex, age, body weight, ethnicity, and comorbidities (hypertension, coronary heart disease, heart failure, diabetes, chronic kidney disease, chronic pulmonary disease, liver disease, anemia, malignant cancer). CKD, chronic kidney disease; SHR, Stress hyperglycemia ratio.

## 4 Discussion

This study represents the novel investigation into the relationship between SHR and the likelihood of developing AKI in patients with sepsis. The RCS curve revealed a non-linear positive association between SHR and the odds of SA-AKI, with a cut-off value identified at 1.55. Logistic regression analysis confirmed that SHR >1.55 was significantly associated with an increased likelihood of SA-AKI. These findings were consistent across subgroup and sensitivity analyses, suggesting the robustness of the results. Given the widespread availability of blood glucose and HbA1c testing and the ease of SHR calculation, SHR could potentially serve as a convenient and effective predictor for SA-AKI.

Stress hyperglycemia is characterized by a marked elevation in blood glucose levels triggered by physiological or pathological stress. Its development is influenced by various complex physiological responses, including hyperproliferation, activation of the sympathoadrenal system, counter-regulatory hormones (e.g., catecholamines, cortisol), and pro-inflammatory cytokines (e.g., TNF-α), which together create a vicious cycle (23–26). By incorporating HbA1c, SHR adjusts for the confounding effects of chronic glucose levels, making it a more precise measure. Recently, SHR has gained recognition as a biomarker significantly associated with increased mortality in various cardiovascular diseases (11, 12, 27, 28). This association may be explained by mechanisms such as endothelial dysfunction and oxidative stress induced by stress hyperglycemia (6). Moreover, hyperglycemia is associated with decreased nitric oxide bioavailability, increased coagulation, and reduced fibrinolytic activity, collectively promoting a prothrombotic state and higher mortality risk (29, 30). Additionally, recent studies have suggested that stress hyperglycemia can stimulate the production of mitochondrial reactive oxygen species (ROS) and advanced glycation end products (AGEs), leading to epigenetic modifications, such as DNA methylation and post-translational histone modifications (31). These epigenetic changes can affect gene expression, and even short-term stress hyperglycemia may have lasting effects on long-term cardiovascular health, a phenomenon referred to as “metabolic memory” (31).

Research indicates that nearly 10% of ICU patients experience stress hyperglycemia (32), an adaptive metabolic response to acute stress (33, 34). However, evidence also suggests that stress hyperglycemia may lead to poorer outcomes in critically ill patients. A study by Mamtani et al., involving 739,152 critically ill patients, demonstrated a significant association between stress hyperglycemia and higher ICU mortality rates, as well as prolonged ICU-LOS (35). Similarly, Zhang et al. identified elevated SHR as an independent risk factor for ICU mortality in a cohort of 3,887 critically ill patients (OR 2.92, 95% CI 2.14–3.97, *P* < 0.001) (36). Sepsis, a common cause of critical illness, often induces stress states due to abnormal immune activation and massive cytokine release (37), leading to significant blood glucose fluctuations. In line with previous studies (16, 17), our research also found that higher SHR levels were associated with increased mortality and longer hospital stays among patients with sepsis. SHR has been proposed as a potential indicator of disease severity in critically ill populations (7, 27). Our study further supports this, showing a significant association between increased SHR levels and higher SOFA scores, which reflect the extent of organ dysfunction in sepsis (*P* < 0.001) (38). However, no significant differences were found in the staging of SA-AKI (*P* > 0.05). Additionally, patients with sepsis exhibiting elevated SHR were more likely to require mechanical ventilation or vasopressor support, highlighting the relationship between high SHR levels and more severe clinical interventions.

The link between stress hyperglycemia and SA-AKI remains unclear, though studies have examined the relationship between stress hyperglycemia and AKI in other contexts. For example, Gao et al. found that in patients with acute myocardial infarction (AMI) and diabetes, each one-unit increase in SHR was associated with a 218% higher odds of developing AKI (OR 3.18, 95% CI 1.99–5.09, *P* < 0.001) (39). Another study identified a U-shaped relationship between SHR and AKI in individuals with congestive heart failure, with an inflection point at 0.98 (40). Moreover, the analysis revealed variations across subgroups, irrespective of the presence of diabetes or CKD (40). This study is the first to establish a significant correlation between elevated SHR levels and the likelihood of developing AKI in patients with sepsis. The differential performance of SHR in diabetic and non-diabetic populations highlights the complex interplay between chronic hyperglycemia and AKI risk. While no significant differences were observed using the 1.55 cutoff, a weaker association between SHR and SA-AKI risk was found in diabetic subgroup. This may be attributed to elevated chronic glucose levels in diabetes, which reduce the magnitude of SHR changes during acute hyperglycemia, as well as renal adaptation to glucose fluctuations and the influence of comorbidities such as hypertension and cardiovascular diseases. These factors likely diminish the direct impact of acute hyperglycemia on AKI risk in diabetic patients. Despite HbA1c correction for chronic hyperglycemia, the intrinsic effects of diabetes cannot be fully eliminated. The decrease in statistical ability caused by a smaller subgroup sample size may also cannot be ignored. Future research should aim to address these limitations to further validate the findings.

The mechanisms by which stress hyperglycemia contributes to adverse outcomes and the development of AKI in patients with sepsis are multifaceted. First, stress hyperglycemia exacerbates the stress response during sepsis, inducing oxidative stress and endoplasmic reticulum stress, which together drive pathological changes in kidney tissue (41–46). Second, hyperglycemia-induced metabolic alterations impair endothelial function. This occurs through increased mitochondrial ROS production, free fatty acid secretion, and disruptions in endothelial nitric oxide synthase (eNOS) and prostacyclin synthase activity (47, 48). These changes compromise endothelial integrity and microvascular function, leading to increased vascular permeability and capillary leakage, which negatively impacts organ perfusion, including that of the kidneys. Third, hyperglycemia promotes atherosclerosis, causing vascular narrowing (49) that reduces renal perfusion and raises the risk of cardiovascular events such as myocardial infarction and stroke, further diminishing renal blood flow. This interplay between heart and kidney dysfunction is referred to as “cardiorenal syndrome” (CRS) (50). Lastly, stress hyperglycemia facilitates non-enzymatic glycation of platelet glycoproteins, contributing to platelet activation and an elevated risk of thrombosis (51). These mechanisms collectively contribute to the poor prognosis and increased incidence of AKI in patients with sepsis.

Our findings hold potential positive value for further guiding the clinical treatment of sepsis patients. Currently, sepsis management strategies emphasize early risk identification and organ function preservation to improve prognosis. The high SHR group (>1.55) showed an 11% higher incidence of SA-AKI compared to the low SHR group, suggesting SHR’s potential as an early warning biomarker for SA-AKI. Notably, septic patients complicated with AKI not only face elevated mortality risks but also impose substantial burdens on healthcare systems, underscoring the clinical-economic significance of early high-risk population identification (3, 5). These findings propose that implementing intensive monitoring (e.g., enhanced urinary output surveillance and frequent renal function assessments) for patients with elevated SHR at admission may surpass conventional approaches. This stratified management strategy could optimize medical resource allocation while enabling high-risk patients to benefit from preventive interventions through early detection. Furthermore, initiating renal protection protocols during early sepsis diagnosis for high-SHR cohorts, including hemodynamic optimization, avoidance of nephrotoxic agents, and dynamic glucose control, may mitigate the progression risks of AKI, consequently reducing requirements for renal replacement therapy and secondary complications such as infections and multi-organ failure (52–54). We believe future high-quality prospective multicenter studies will be crucial to validate these findings and advance the clinical application of SHR in sepsis management.

Despite offering novel insights, this study has limitations that warrant cautious interpretation. First, as a retrospective analysis, it does not establish a causal relationship between SHR and SA-AKI development. Second, certain potential confounders, such as hemoglobinopathies, hypothyroidism, and the use of iron supplements or erythropoietin, which can influence HbA1c levels, were not excluded (55–57). Third, although logistic regression was used to adjust for several confounders, residual unknown factors may still affect the reliability of the findings. Fourth, since the identification of SA-AKI cases in this study was solely based on serum creatinine levels, we might have missed some SA-AKI cases that presented primarily with anuria or oliguria. Therefore, the applicability of our findings to SA-AKI patients without elevated creatinine levels remains uncertain. Accurately determining the temporal sequence between blood glucose/HbA1c measurements and AKI onset is challenging. We cannot guarantee that creatinine measurements were obtained immediately at AKI onset, and using the timing of creatinine measurement as the AKI diagnosis time may introduce bias. Finally, the single-center design limits the generalizability of the results, despite the large sample size provided by the MIMIC database. Therefore, multi-center and prospective studies are essential in order to confirm these findings.

## 5 Conclusions

The current study revealed a non-linear positive relationship between SHR and the likelihood of AKI in patients with sepsis, with SHR > 1.55 significantly associated with a higher likelihood of developing SA-AKI. As a novel biomarker, SHR shows promise for aiding in the early detection and prevention of SA-AKI, potentially improving patient outcomes.

## Availability of data and materials

The study utilized data from the publicly accessible MIMIC-IV (v2.2) database, available at https://physionet.org/content/mimiciv/2.2/. Raw data and analysis code can be obtained from the corresponding author upon request.

## Data Availability

The original contributions presented in the study are included in the article/supplementary material. Further inquiries can be directed to the corresponding authors.
